# Design of Laser Photothermal Conversion Membranes Based on Fluorinated Graphene

**DOI:** 10.3390/membranes12020135

**Published:** 2022-01-23

**Authors:** Junyu Piao, Keding Li, Yong Zhang, Long Zhang

**Affiliations:** 1 Institute of Chemical Materials, China Academy of Engineering Physics, Mianyang 621999, China; piaojunyu@caep.cn; 2 National Defense Science and Technology College, Southwest University of Science and Technology, Mianyang 621010, China; kdiaser007@163.com

**Keywords:** fluorinated graphene, 2D materials, membrane, photothermal conversion, surface functionalization

## Abstract

Laser photothermal-conversion membranes have great potential applications in many different fields, including laser ignition. However, the demand for real-time, high heat output calls for an extra heat-releasing pattern other than the traditional luminous energy–thermal, energy-conversion mechanism. Herein, it was found that fluorinated graphene (FG) was a promising candidate for laser photothermal conversion due to the extra chemical energy–thermal, energy-conversion process, which originated from a self-redox reaction under laser irradiation. Moreover, an easy sonochemical, exfoliation–filtration protocol was provided for the preparation of the fluorinated, graphene-based, free-standing membranes. In brief, FG flakes were arranged into flower-like patterns and formed freestanding, carpet-like membranes with layered structures with the filtration of FG suspension, which was obtained from exfoliating fluorographite in N-methylpyrrolidone. Furthermore, this contribution also revealed that modifying the FG membranes with polytetrafluoroethylene (PTFE) was helpful for improving the photothermal-conversion properties. With the construction of the FG/PTFE composited structure, higher heat output could be achieved when a laser pulse is applied to the composite membranes. This work revealed the great potential of fluorinated graphene in laser photothermal conversion, and provided an alternative route of introducing a chemical energy–thermal, energy-conversion process for achieving high heat output under laser irradiation.

## 1. Introduction

Laser ignition, which usually refers to the combustion or explosion triggered by a short pulse of laser, has been used in a wide variety of applications, including internal combustion engines, pyrotechnics, and rocket motors [1,2,3]. As a light source, laser is able to concentrate extremely high energy over a very small irradiation area. Laser photothermal-conversion membranes have recently been identified as promising lase, energy-conversion devices for various applications, such as biomedicines, laser ignition, and sensing [3,4,5,6,7]. As opposed to widely-used solar, photothermal-conversion membranes, more attention needs to be paid to the rapid, real-time thermal response and high heat output under laser irradiation for laser photothermal-conversion membranes.

Commonly, photothermal conversion is mainly achieved by plasmon resonance or non-radiative transition of surface carriers [8,9,10]. When laser irradiation is applied, the surface carriers will be excited from the ground state to the lowest-excited singlet state, and then the plasmon resonance or non-radiative relaxation pathway will decay back to the ground state and produce a photothermal effect [11,12]. Thus, the heat output capacity of laser photothermal-conversion membranes are confined to the limited number of carriers over the small irradiation area. Therefore, it is of great importance to develop a new method for improving the heat-output performance in the laser photothermal-conversion process.

Although good light absorption is a prerequisite for good photothermal-conversion capability, it is still not enough for laser photothermal-conversion membranes to achieve high heat output under laser irradiation [13,14,15,16]. For instance, although graphene has high absorbance over a wide range of wavelengths due to its characteristic 2D structure composed of sp^2^ carbons [13,17], its heat-releasing performance under laser irradiation is still far from being ideal, which is partially related to its high thermal conductivity [6,14,17,18]. Interestingly, it was reported that graphene oxide (GO) had more superior photothermal properties when a laser pulse was applied to it [14,19]. As opposed to graphene, GO possesses some oxidizing groups, such as carboxyl, epoxy, and hydroxyl groups [20,21], which will react with graphene interlayers under laser irradiation and release extra heat [14,15]. Therefore, introducing a chemical energy–thermal, energy-conversion process is an effective way to improve the heat-output properties of the photothermal conversion membranes under laser irradiation.

Fluorinated graphene (FG), which can be considered as a partially fluorinated graphene sheet [22,23], is a promising two-dimensional material for laser photothermal conversion. Firstly, the high light absorbance of FG will facilitate laser absorption and contribute to heat release through non-radiative transition [23,24,25,26,27,28,29,30]. Secondly, the reductive carbon, the oxidative fluorine, and the CF_x_ free radicals generated at high temperatures will lead to self-redox reactions under laser irradiation, which will result in extra heat release [2,30]. Therefore, it is reasonable to design laser photothermal-conversion membranes with superior heat output based on FG. Unfortunately, most works about FG in the field of laser photothermal conversion only focused on the high near-infrared (NIR) absorption and the strong adsorption energy between energetic materials [26,27,28,29,30], and little work has been engaged in the characteristic self-redox properties of FG and the significance of introducing an additional heat-releasing process, other than photothermal conversion.

In this contribution, we provide an easy preparation protocol of freestanding, two-dimensional FG membranes. When being used as laser photothermal-conversion membranes, FG membranes exhibit considerable heat output under laser irradiation due to the introduction of an oxidative F element on the graphene interlayers. Moreover, it is found that modifying the FG membranes with polytetrafluoroethylene (PTFE), and constructing an FG/PTFE-composited structure, will further improve the photothermal properties of the FG-based membranes. Accordingly, higher laser-induced temperature rises will be achieved for the FG/PTFE-composited membranes. This work reveals the great potential of fluorinated graphene in laser photothermal conversion, and provides a new perspective for introducing the chemical energy–thermal, energy-conversion process for achieving high heat output under laser irradiation.

## 2. Materials and Methods

All the chemicals were of analytical purity and were purchased from Sinopharm Chemical Reagent Co., Ltd. (Shanghai, China). The fluorine content of the commercial fluorographite powder was 62% by mass. Accordingly, the C:F molar ratio was close to 1:1.

FG was prepared through a sonochemical method [31]. Briefly, 1 g of fluorographite powder was added into 200 mL of N-methylpyrrolidone (NMP). After refluxing at 60 °C for 2 h, the mixed suspension was treated with an Branson S-250D (Branson, USA) ultrasonic cell crusher at around 80 W for 2 h. FG suspension was obtained after the sonication process. The fluorine content of FG was equal to that of the commercial fluorographite powder (62%).

An FG membrane was prepared with a standard filtration process based on a static deposition mechanism [32,33]. Briefly, FG suspension, containing 20 mg of FG, was used for filtration, and one freestanding FG membrane was obtained through a self-assembly process on the top side of the filter paper (diameter: Φ50 mm, pore size: 0.2 µm). The FG membrane could be peeled off from the filter paper after drying. Similarly, a reduced graphene oxide (rGO) membrane was prepared by filtering the rGO/ethanol suspension, and one rGO membrane contained 20 mg of rGO. The diameter of the membranes was equal to the filter paper (Φ50 mm), and the thickness of the membranes was around 5 µm. Accordingly, the surface density of the membranes was around 1 mg/cm^2^.

FG/PTFE-composited membranes were also prepared through a similar filtration process, while FG/PTFE mixed suspension was used for filtration, and the total weight of FG and PTFE was 20 mg. The FG/PTFE mixed suspension was obtained by simply adding a specific amount of PTFE powder into the prepared FG suspension with stirring. For the sample of FG-PTFE, the weight ratio of FG and PTFE was 2:1; and for the sample of FG-PTFE double, the weight ratio of FG and PTFE was 1:1.

Scanning electron microscopy (SEM) experiments were recorded on a Hitachi TM4000 (Tokyo, Japan) scanning electron microscope. X-ray diffraction (XRD) patterns were carried out on a Rigaku D/MAX-2500 (Tokyo, Japan) with Cu K_α_ radiation at 50 kV and 250 mA. Thermogravimetry analysis (TGA) was performed on a Mettler-Toledo TGA/DSC3+/1100LF system with a heating speed of 20 °C/min. The wavelength, the beam diameter, and the pulse time for the diode laser system (BWT Beijing Company, Beijing, China) were 450 nm, 105 μm, and 300 ms, respectively. The temperature of the membranes under laser irradiation was measured by an infrared camera (FLIR E60), whose precision was ±2 °C or ±2% (the larger one). The ultraviolet-visible (UV–Vis) absorption spectra were collected with an Agilent Cary 5000 (Santa Clara, USA) spectrophotometer. The laser-induced temperature rise was measured by an FLIR E60 (Wilsonville, USA) infrared camera.

## 3. Results and Discussion

### 3.1. Morphology and Composition

**Scheme 1:**
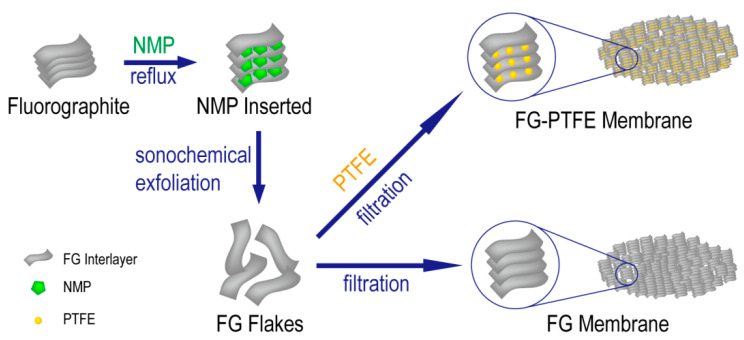
Preparation of FG membranes and FG-PTFE membranes.

**Figure 1:**
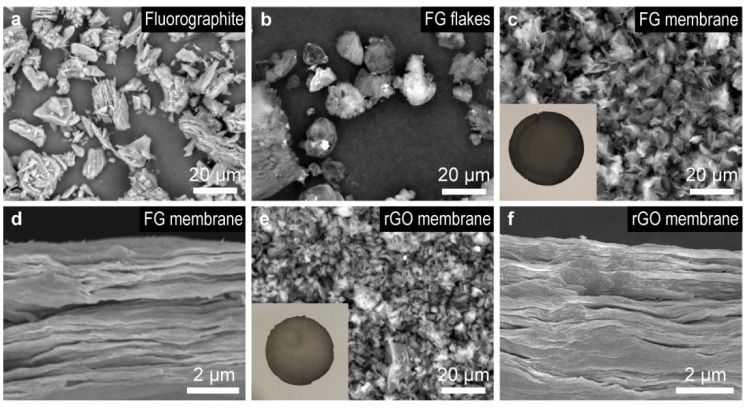
Morphology of fluorographite, FG, and rGO. (**a**) SEM image of fluorographite. (**b**) SEM image of FG flakes. (**c**) SEM image and photograph of an FG membrane. (**d**) SEM cross-sectional image of an FG membrane. (**e**) SEM image and photograph of an rGO membrane. (**f**) SEM cross-sectional image of an rGO membrane.

Owing to the characteristic layered structure, which was similar to graphite (Figure 1a), fluorographite could be used as the raw material for the preparation of FG [31]. Briefly, as shown in Scheme 1, fluorographite powder was first dispersed in NMP and then underwent a reflux process at 60 °C, which would endow NMP molecules with sufficient energy to be inserted into the interlayer of fluorographite. Moreover, FG flakes were obtained during the sonochemical exfoliation process because of the weakened binding force between the two adjacent fluorographite layers (Figure 1b). Considering the mechanism of exfoliation, the structure and the C:F ratio of FG flakes did not change in the preparation process. With the merit of the unique 2D structure [6,33,34], freestanding FG membranes could be easily prepared through a simple filtration process. As shown in Figure 1c,d, FG flakes were arranged into flower-like patterns and formed freestanding, carpet-like membranes with a layered structure. Notably, considering the micrometer level size of the FG flakes, which was much larger than the pore size (0.2 µm), almost all the FG was deposited on the filter paper and formed membranes rather than going to the permeate sides. As a reference sample, rGO membranes were also prepared through a similar filtration process, which were also of a carpet-like morphology with a layered structure (Figure 1e,f).

**Figure 2:**
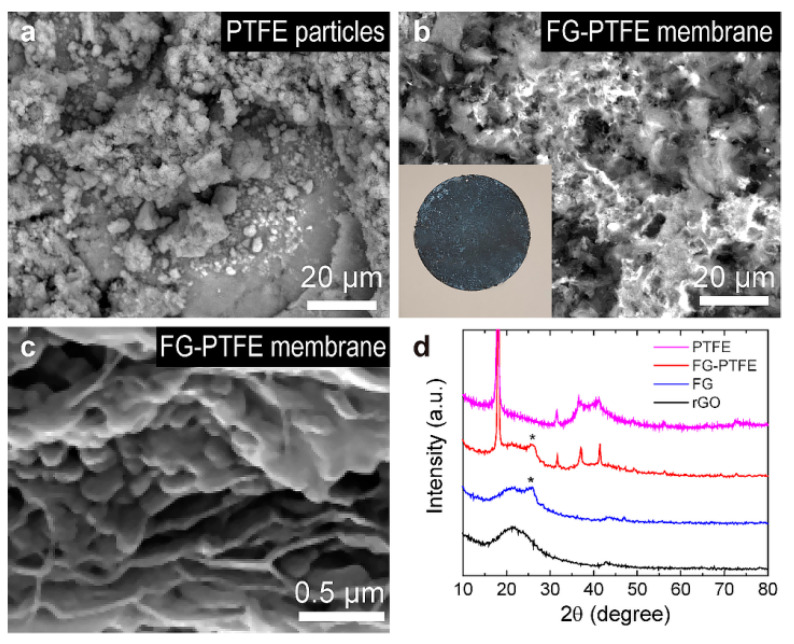
Morphology of PTFE-containing samples, and the XRD patterns. (**a**) SEM image of PTFE particles. (**b**) SEM image and photograph of an FG-PTFE membrane. (**c**) SEM cross-sectional image of an FG-PTFE membrane. (**d**) XRD patterns of rGO, FG, FG-PTFE, and PTFE.

Although PTFE has usually been considered as a chemically-inert material, things changed in the field of energetic materials. Due to the high content of oxidative fluorine (76%), the strongly oxidative CF_x_ free radicals generated at high temperatures, and the high exothermic enthalpy (ΔH) of the fluorinated products [23,34,35,36,37], PTFE could be used as an oxidative additive to modify the heat-output properties of FG-based membranes. Commonly, the explosion/combustion process would provide enough energy for the pyrolysis of PTFE, and the pyrolysis products would act as oxidants and accelerate the explosion/combustion process in return. As shown in Figure 2a, PTFE was of spherical shape, with a micrometer-level diameter. Similarly, FG-PTFE-composited membranes could also be easily obtained by filtrating the mixture of FG and PTFE. As shown in Figure 2b,c, FG-PTFE membranes were also of carpet-like morphology, while the PTFE particles were sandwiched between the FG flakes. The preparation process is also illustrated in Scheme 1.

The composition of the freestanding membranes were studied by X-ray diffraction (XRD) analysis, and the results are exhibited in Figure 2d. A characteristic wide bulge between 15° and 30° could be seen in the XRD patterns of rGO, FG, and FG-PTFE, which revealed a layered structure, similar to graphite [31,38]. Moreover, the asterisked peak around 26° in the patterns of FG and FG-PTFE indicated the existence of sp^3^ carbons due to the formation of C–F bonds in the FG interlayers [39]. Notably, the pattern of FG-PTFE could be regarded as the combination of the patterns of FG and PTFE, which was in good accordance with the sandwich-like structure of FG-PTFE (Figure 2c).

### 3.2. Photothermal Properties

The photothermal-conversion properties of the freestanding membrane were examined with laser-induced, temperature-rise tests. Briefly, when a laser pulse was applied to the photothermal-conversion membrane, such membranes would absorb the laser and convert luminous energy into thermal energy, inducing a drastic increase in temperature. Thus, at the same experimental conditions, higher temperature rises indicated more superior photothermal-conversion properties. Moreover, considering the fact that photons with shorter wavelengths possess higher energy, which is beneficial for laser ignition, a 450 nm laser was used in the laser-induced, temperature-rise tests instead of the widely-used NIR laser.

**Figure 3:**
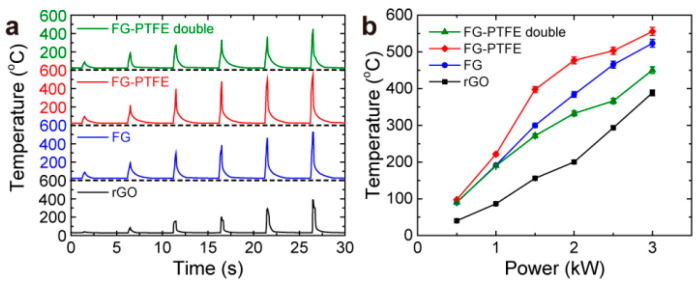
Results of the laser-induced, temperature-rise tests. (**a**) Temperature profiles when the laser pulse was applied. (**b**) Laser-induced temperature rise at different laser powers. The error bars were plotted according to the precision of the thermometer at each temperature.

The results of the laser-induced, temperature-rise tests are shown in Figure 3. In order to characterize the response of the membranes to laser irradiation with different energy densities, laser pulses with a peak power from 0.5 kW to 3.0 kW were used in the tests. Notably, for all the samples, a sharp temperature-rise peak arose when the laser pulse was applied, and higher temperature rises were obtained with the increase in the energy density of the laser. Although oxidation of membranes with air oxygen occurred during the laser irradiation, considering the almost-unchanged oxygen concentration and the fixed surface density of the membranes, it was inferred that the influence of air oxygen should be regarded as a nearly constant factor for each of the cases. Since potential errors from the machine resolution were ±2 °C or ±2% (the larger one), the precision of the temperature-rise tests was adequate for characterizing the heat-output performance of these membranes. Notably, it was found that the temperature rise of FG was much higher than that of rGO at each laser power, which proved that the FG membrane possessed more superior photothermal properties than rGO. Interestingly, an even higher temperature rise was obtained for the sample of FG-PTFE, revealing that the introduction of PTFE, and the construction of the FG/PTFE-sandwiched structure, would help to improve the photothermal properties of FG-based, photothermal-conversion membranes. However, when the amount of PTFE was doubled (denoted as FG-PTFE double), the laser-induced temperature rise would decrease, which indicated that introducing an excess amount of PTFE was destructive for designing membranes with good photothermal properties.

### 3.3. Mechanism Research

**Figure 4:**
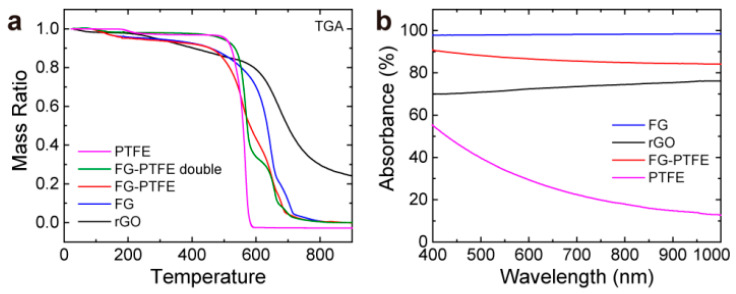
TGA and UV-Vis absorption curves. (**a**) TGA curves of different samples. (**b**) UN-Vis absorption curves of different samples.

In order to understand the relationship between the photothermal properties of each sample and their components, we carried out thermal and optical analyses on these samples. As the circumstances of laser ignition were more similar to air atmosphere than argon atmosphere, we decided to do TGA tests in air atmosphere. Figure 4a shows the TGA curves that were tested in air atmosphere, and the corresponding 5% weight loss temperatures are shown in Table 1 as a reflection of thermal stability. For the rGO sample, over 20% of the initial weight remained, even at 900 °C, revealing that rGO was difficult to oxidize. On the contrary, FG lost almost all the weight when the temperature was higher than 800 °C, proving that FG could be oxidized and release heat more easily than rGO. The superior thermal properties of FG could be attributed to the self-redox reaction, which originated from the existence of reductive carbon, oxidative fluorine, and the CF_x_ free radicals generated at high temperatures [2,30]. As a result, higher laser-induced temperature rises were obtained for FG compared with rGO. Moreover, as shown in Table 1, the 5% weight loss temperature of FG-PTFE was 213.8 °C, which was lower than that of rGO and FG, revealing that introducing PTFE as an oxidative additive was an effective way to modify the heat-output properties of FG membranes. However, for the sample of FG-PTFE double, the weight was lost in two steps, which represented the decomposition of PTFE and the oxidation of FG, respectively. Thus, when an excess amount of PTFE was introduced, redundant PTFE did not contribute to the oxidation of FG, which led to a decrease in energy density. Accordingly, a relatively high 5% weight loss temperature (393.0 °C) was achieved for he FG-PTFE double sample, which was even higher than FG (Table 1). Therefore, proper amounts of PTFE in the FG/PTFE-composited membrane was of great importance for achieving superior photothermal properties.

The light absorbance of each sample were analyzed with the UV-Vis absorption spectra. As shown in Figure 4b, FG exhibited excellent light absorbance (around 98%) between 400 nm and 1000 nm, which was higher than that of rGO (70-76%). Thus, luminous energy could be utilized more efficiently for FG than rGO when the laser pulse was applied to the membranes. Although FG has a similar chemical formula to PTFE ((CF)_x_ for FG and (CF_2_)_x_ for PTFE), the structure of these two samples are quite different (2D layer of six-member rings for FG and linear chains for PTFE), which leads to different properties in light absorbance. Since PTFE was not a good light absorber (lower than 60%), introducing an excess amount of PTFE led to unacceptably low light absorbance. Accordingly, the construction of the FG/PTFE-composited structure with proper light absorbance was necessary for achieving good photothermal properties. Encouragingly, FG-PTFE possessed good light absorbance (even higher than rGO), which was also helpful for improving photothermal performance.

## 4. Conclusions

Good light absorption is not a sufficient condition to achieve high heat output, especially in the case of laser ignition. Therefore, it is of great importance to develop a new strategy to improve the heat-output performance of laser photothermal-conversion membranes. In this work, it was proposed that introducing a chemical energy–thermal, energy-conversion process is an effective way to obtain optimized heat output under laser irradiation. Accordingly, fluorinated graphene (FG) was shown to be a promising material for laser photothermal conversion, especially for laser ignition, due to the presence of the chemical energy–thermal, energy-conversion process, which originated from the self-redox reaction under laser irradiation. Moreover, freestanding FG membranes were prepared through a simple exfoliation/filtration protocol, which showed high laser-induced temperature increases when a laser pulse was applied. Furthermore, it was also found that modifying the FG membranes with PTFE and constructing FG/PTFE-composite structures contributed to the further improvement of the laser photothermal-conversion properties. This work provides a new perspective for the realization of high heat output under laser irradiation, and reveals the great potential of FG in the field of laser photothermal conversion.

## Figures and Tables

**Table 1:** The 5% weight loss temperatures of different samples.

Sample	rGO	FG	FG-PTFE	FG-PTFE Double	PTFE
5% weight loss temperature (°C)	288.7	268.7	213.8	393.0	503.6

## Data Availability

Not applicable.
